# Optimization of Primary Human Bronchial Epithelial 3D Cell Culture with Donor-Matched Fibroblasts and Comparison of Two Different Culture Media

**DOI:** 10.3390/ijms24044113

**Published:** 2023-02-18

**Authors:** Julian Maurer, Thorsten Walles, Cornelia Wiese-Rischke

**Affiliations:** University Clinic for Cardiac and Thoracic Surgery, Otto-von-Guericke-University Magdeburg, D-39120 Magdeburg, Germany

**Keywords:** 3D airway model, human primary bronchial epithelial cells, fibroblasts, co-culture

## Abstract

In vitro airway models are increasingly important for pathomechanistic analyses of respiratory diseases. Existing models are limited in their validity by their incomplete cellular complexity. We therefore aimed to generate a more complex and meaningful three-dimensional (3D) airway model. Primary human bronchial epithelial cells (hbEC) were propagated in airway epithelial cell growth (AECG) or PneumaCult ExPlus medium. Generating 3D models, hbEC were airlifted and cultured on a collagen matrix with donor-matched bronchial fibroblasts for 21 days comparing two media (AECG or PneumaCult ALI (PC ALI)). 3D models were characterized by histology and immunofluorescence staining. The epithelial barrier function was quantified by transepithelial electrical resistance (TEER) measurements. The presence and function of ciliated epithelium were determined by Western blot and microscopy with high-speed camera. In 2D cultures, an increased number of cytokeratin 14-positive hbEC was present with AECG medium. In 3D models, AECG medium accounted for high proliferation, resulting in hypertrophic epithelium and fluctuating TEER values. Models cultured with PC ALI medium developed a functional ciliated epithelium with a stable epithelial barrier. Here, we established a 3D model with high in vivo–in vitro correlation, which has the potential to close the translational gap for investigations of the human respiratory epithelium in pharmacological, infectiological, and inflammatory research.

## 1. Introduction

Respiratory diseases are among the leading causes of death worldwide [1]. Lung infections, lung cancer, and chronic obstructive pulmonary disease (COPD) account for one-sixth of all deaths. The global burden of lung disease is increasing steadily—well before the onset of SARS-CoV-2 and the COVID-19 pandemic. Biomedical research tries to identify the underlying disease mechanisms.

The respiratory epithelium plays a pivotal role in disease pathogenesis and progression [2,3]. In addition, for the treatment of lung diseases as well as other organ diseases, the respiratory epithelium, with its size of 100 square meters, represents a promising approach for drug delivery. Drug delivery via the respiratory system is influenced by many anatomical and physiological factors such as lung morphometry, respiratory patterns, fluid dynamics, particle properties, etc. [4].

During the last two decades, these questions have been investigated in many methodologically different research approaches. While important insights into the pathomechanism of acute and chronic lung damage could be identified in small animal models, large animal models have been established for drug development, not least because of their closer proximity to the anatomical and physiological situation in the human lung [4,5,6,7,8]. However, the usefulness of large animals for research is limited by the high costs associated with this type of research, and the incomplete understanding of disease mechanisms in the species used compared to rodents [9]. 

In contrast to animal experiments, in vitro models from human cell lines or primary cells are significantly cheaper and may allow high-throughput research approaches [5]. Depending on the model properties, they can reproduce the pathomechanisms in human airways [10,11]. For example, it was shown very early that the infection of the human respiratory system with the SARS coronavirus can only be reproduced in realistically constructed in vitro models, with a pseudostratified epithelium with polarized cells [12]. The requirements for basic and applied research in the course of the SARS-CoV-2 pandemic have shown the limitations of the existing in vitro airway models and contributed to their further development [13,14,15].

Tissue engineering has made considerable progress in generating 3D tissue models for basic and applied research. For the generation of human airway models, cancer-derived epithelial cell lines have been applied, though many cell lines are not capable of complete epithelial differentiation [16,17,18]. In contrast, primary airway epithelial cells are able to fully differentiate into a mature epithelium, but cultivation and differentiation of these cells remains challenging. Their limitations further include a limited number of cell duplications, limited availability of donor tissue and high donor variability.

In order to induce epithelial differentiation, air-liquid-interface (ALI) culture methods are used [19,20,21]. Current models fail to mimic the complex in vivo situation of the airway consisting of bronchial epithelium and a stromal component composed of a lamina propria with fibroblasts. Further, the co-cultivation with fibroblasts has been already shown to increase the differentiation potential of epithelial cells [22,23]. Previous studies used collagen-coated transwell inserts [24] or multilayered cultures with more than one scaffold [25] to enable co-cultivation, but direct cell-cell contacts are still missing. Although, formation of 3D human airway models cultivated with either epithelial growth [23,26] or differentiation media [19,21] have been reported, the cell culture medium plays an important role in tissue differentiation. Therefore, the aim of this study was to generate a human 3D airway tissue model that resembles native tissue with pseudostratified epithelium consisting of ciliated cells with actively beating cilia, mucin-secreting goblet cells, and basal cells. Such a model would be composed not only of an epithelial but also a stromal cell compartment with a biological scaffold. Therefore, in this study, we co-cultured primary bronchial epithelial cells with donor-matched bronchial fibroblasts on a decellularized porcine small intestinal submucosa (SIS) as collagen scaffold. The effect of two different media on epithelial differentiation was analyzed.

## 2. Results

### 2.1. Optimized Cell Isolation and Expansion

**Figure 1:**
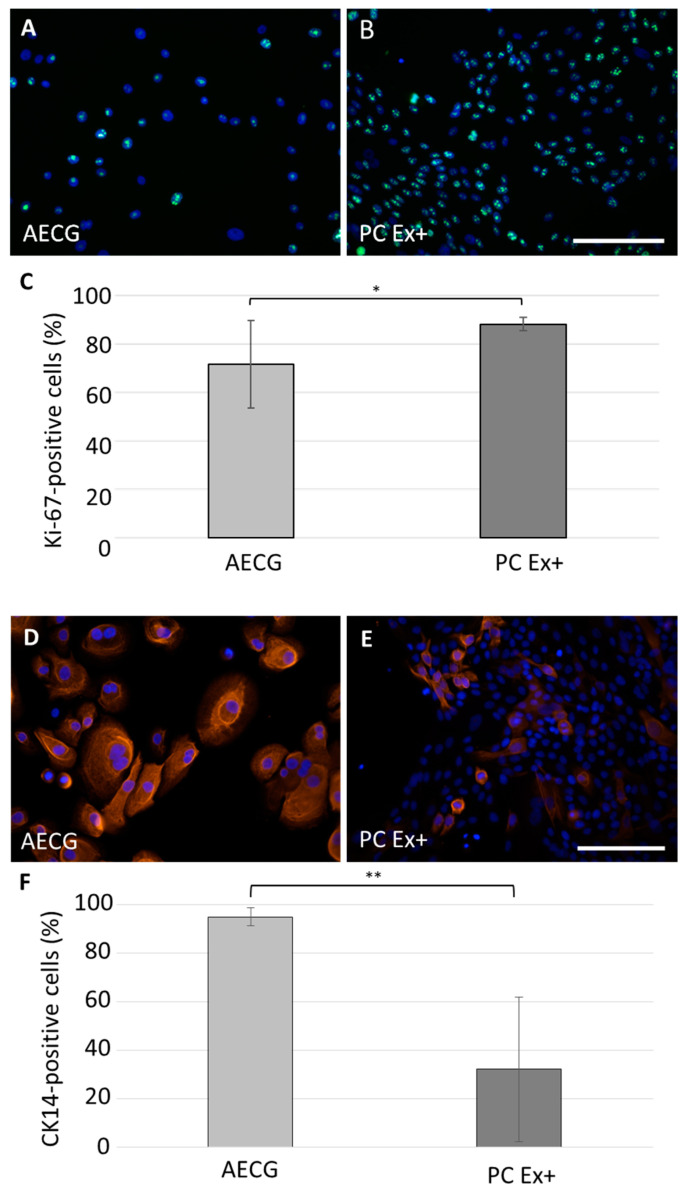
Expansion of primary human bronchial epithelial cells (hbEC). hbEC were expanded either in airway epithelial cell growth (AECG) or PneumaCult ExPlus (PC Ex+) medium and characterized with immunofluorescence staining in passage 1. (**A**–**C**) Immunofluorescence staining against Ki-67 (green) on hbEC cultivated with (**A**) AECG and (**B**) PC Ex+ medium and (**C**) determination of the percentage of Ki-67-positive cells (AECG (light grey, n = 5 from three donors); PC Ex+ (dark grey, n = 4 from three donors). (**D**–**F**) Immunofluorescence staining against cytokeratin 14 (CK14, red) on hbEC cultivated with (**D**) AECG or (**E**) PC Ex+ medium and (**F**) evaluation of the percentage of CK14-positive cells (AECG (light grey) and PC Ex+ (dark grey), n = 7 each from five donors). Nuclei were stained with DAPI (blue). Cell culture in AECG medium resulted in increased cell size. Scale bar: 100 µm. Shown are the mean average with standard deviation. * *p* < 0.05; ** *p* < 0.001.

In order to isolate and expand bronchial epithelial cells, two different culture media were compared: airway epithelial cell growth (AECG) and PneumaCult ExPlus (PC Ex+) medium. Following plating of tissue biopsies, ring-shaped outgrowths around the tissue pieces were observed for all used biopsies after three to seven days with both the AECG and PC Ex+ medium. In AECG medium, epithelial cells were big and formed extensions (Figure S1). In contrast, epithelial cells in PC Ex+ medium were small and polygonal (Figure S1). From all donors, bronchial epithelial cells and fibroblasts could be separately isolated and expanded. To investigate the influence of the two different media on epithelial cell proliferation, immunofluorescence stainings against Ki-67 at passage 1 were performed and quantified (Figure 1A–C). With the AECG medium 72 ± 18% and with PC Ex+ medium 88 ± 3% of cells were Ki-67 positive (*p* = 0.0317) (Figure 1C). Accordingly, cell yield after the first passage was significantly higher with PC Ex+ medium compared to AECG medium (8.9 × 10^6^ cells ± 4.8 × 10^6^ cells vs. 3.4 × 10^6^ cells ± 1.26 × 10^6^ cells, *p* = 0.0043, from six different donors). To evaluate the basal cell compartment, we performed immunofluorescence stainings against p63 (pan-basal epithelial cell marker) (Figure S2) and cytokeratin 14 (CK14, for subpopulation of basal cells) (Figure 1D–F). Almost all cells were p63 positive under both culture conditions (Figure S2). Immunostaining against CK14 showed significant more CK14-positive cells when cultivated with AECG medium (95 ± 4%) compared to PC Ex+ medium (32 ± 30%) (*p* = 0.0006) (Figure 1F). This indicates that different basal cell populations are propagated dependent on the cultivation medium used. Our data further showed a higher proliferation and cell yield when cultivated with PC Ex+-medium.

### 2.2. Morphological Differentiation of 3D Models

**Figure 2:**
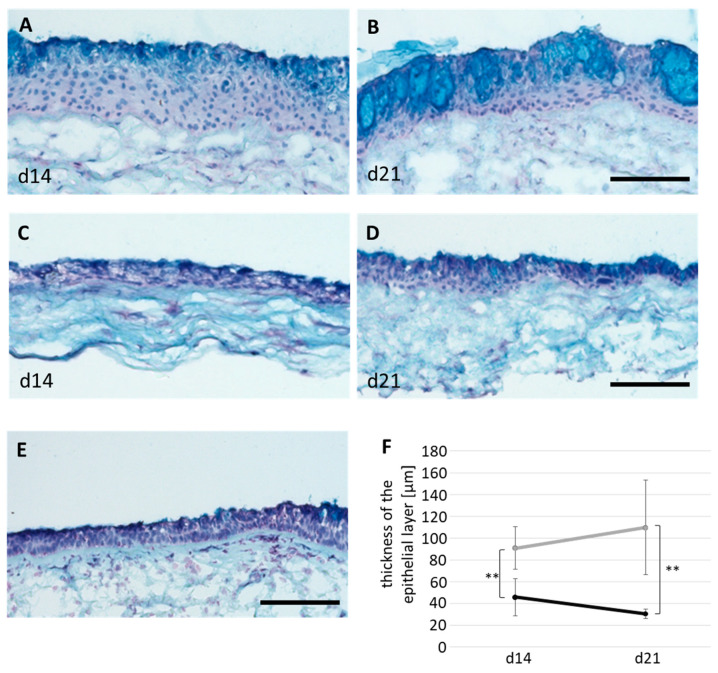
Histological characterization of 3D airway tissue models. Models were cultivated for 14 and 21 days, respectively, at air-liquid-interface (ALI) with either AECG or PneumaCult ALI (PC ALI) medium. (**A**,**B**) Alcian blue staining of cross sections of 3D models cultivated with AECG for (**A**) 14 and (**B**) 21 days (n = 10 each from four donors). (**C**,**D**) Alcian blue staining of cross sections of 3D models cultivated with PC ALI for (**C**) 14 and (**D**) 21 days (n = 6 each from four donors). (**E**) Alcian blue staining of native bronchus (control). (**F**) Determination of the epithelial layer thickness (AECG (light grey), n = 9; PC ALI (dark grey), n = 6, both from four donors). Scale bar: 200 µm. Shown are the mean average with standard deviation. ** *p* < 0.01.

**Figure 3:**
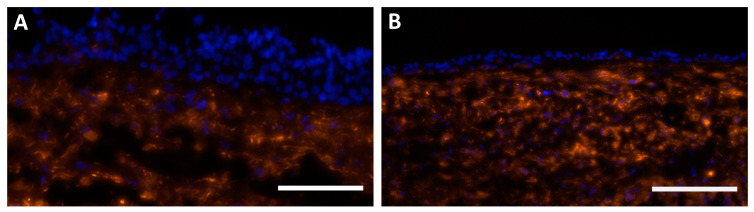
Characterization of the stromal compartment. Immunofluorescence staining against vimentin (orange) on cross sections of 3D models after 21 days of cultivation with (**A**) AECG (n = 9 from four donors) and (**B**) PC ALI medium (n = 7 from four donors). Nuclei were stained with DAPI (blue). Scale bar: 200 µm.

In all 3D tissue models, human bronchial fibroblasts (hbFb) migrated into the biological scaffold and hbEC formed a cell layer at the surface (Figure 2A–D). 3D models cultivated with AECG medium showed a hyperplastic epithelium and non-directed mucus secretion, resulting in intraepithelial mucus cysts (Figure 2A,B). The epithelial layer thickness was 91 ± 20 µm at 14 days and 110 ± 44 µm at 21 days of ALI cultivation, respectively (Figure 2F). Models cultivated with PneumaCult ALI (PC ALI) medium developed a thinner, pseudostratified epithelium with apical mucus secretion (Figure 2C,D) and resembled the morphology of native bronchus (Figure 2E). These models reached an epithelial layer thickness of 46 ± 17 µm at 14 days, which decreased to 31 ± 4 µm at 21 days, respectively (Figure 2F). In addition, vimentin staining confirmed a successful co-culture with hbFb which migrated into the SIS scaffold (Figure 3A,B). All 3D models developed a basal cell layer with p63-positive basal cells (Figure S3). Thus, our data indicate a medium-dependent morphological differentiation of the 3D models. Only 3D models with PC ALI medium developed a pseudostratified epithelium with physiological thickness.

### 2.3. Mucus Production

**Figure 4:**
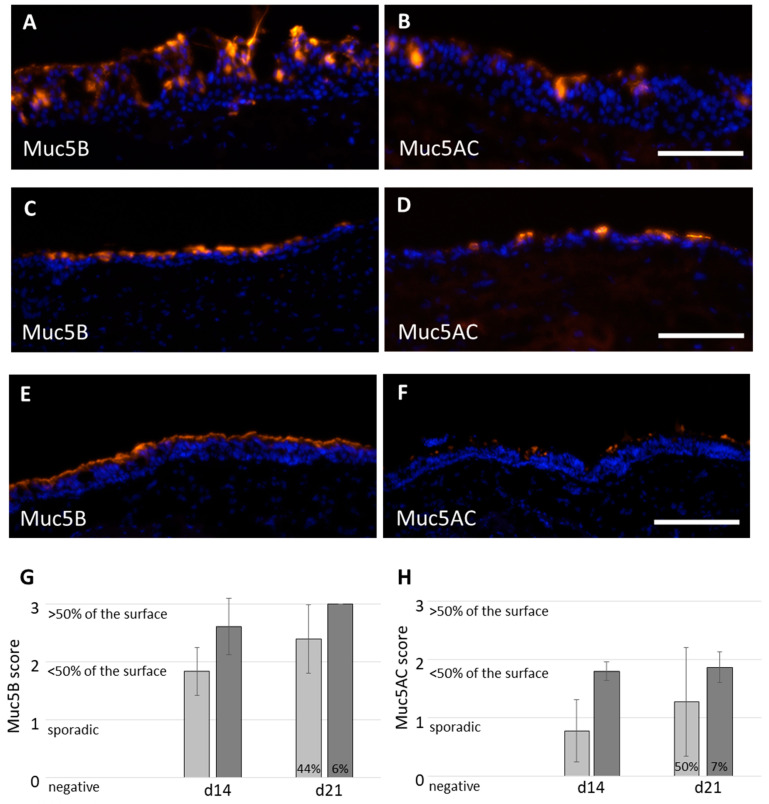
Mucus production in 3D airway tissue models. Immunofluorescence stainings of cross sections of 21 days old ALI models against Muc5B (orange, (**A**,**C**,**E**)) and Muc5AC (orange, (**B**,**D**,**F**)) cultivated with ((**A**,**B**), n = 9 from four donors) AECG and ((**C**,**D**), n = 6 from four donors) PC ALI medium. (**E**,**F**) staining of native bronchus (control). The amount of Muc5B (**G**) and Muc5AC (**H**) was scored (Muc5B: AECG and PC ALI n = 6; Muc5AC: AECG n = 6; PC ALI n = 5, all from four donors) with AECG in light grey bars and PC ALI medium in dark grey bars. The percentage of cross sections containing intraepithelial mucus cysts is shown within the bars at day 21. Nuclei were stained with DAPI (blue). Scale bar: 200 µm. Shown are the mean average with standard deviation.

The immunofluorescence stainings against Muc5B and Muc5AC confirmed mucus production, independent from the culture medium applied (Figure 4A–D). 3D tissue models cultivated with AECG medium showed mainly a non-directional intraepithelial mucus secretion at day 21 (Figure 2B and Figure 4A,B), compared to the apical mucus secretion with PC ALI medium (Figure 2D and Figure 4C,D). The native bronchus also exhibited apical mucus secretion, and more Muc5B than Muc5AC protein was present (Figure 4E,F). The Muc5B and Muc5AC secretion was semi-quantitatively analyzed using a scoring system. Zero points means no secretion, one point means sporadic secretion, and two and three points mean <50% and >50% secretion, respectively. The Muc5B secretion was 1.8 ± 0.4 points at day 14 and 2.4 ± 0.6 points at day 21 for AECG, and 2.6 ± 0.5 points at day 14 and 3.0 ± 0.0 points at day 21 for PC ALI (Figure 4G). Intraepithelial mucus cysts were present in 44% and 6% of the analyzed areas following AECG or PC ALI medium, respectively. Analysis of the Muc5AC secretion resulted in 0.8 ± 0.5 points at day 14, and 1.3 ± 0.9 points at day 21, with AECG medium and 1.8 ± 0.2 points at day 14 and 1.9 ± 0.3 points at day 21 for PC ALI (Figure 4H). For Muc5AC, 50% (AECG) and 6.7% (PC ALI) of the analyzed areas showed intraepithelial mucus cysts. Thus, the secretion of mucins at day 21 showed a higher donor-to-donor variability with AECG medium than with PC ALI medium. The results indicated a higher mucin secretion of both mucus types and less intraepithelial mucus cysts with PC ALI medium.

### 2.4. Differentiation of Apical Cilia with Physiological Function

**Figure 5:**
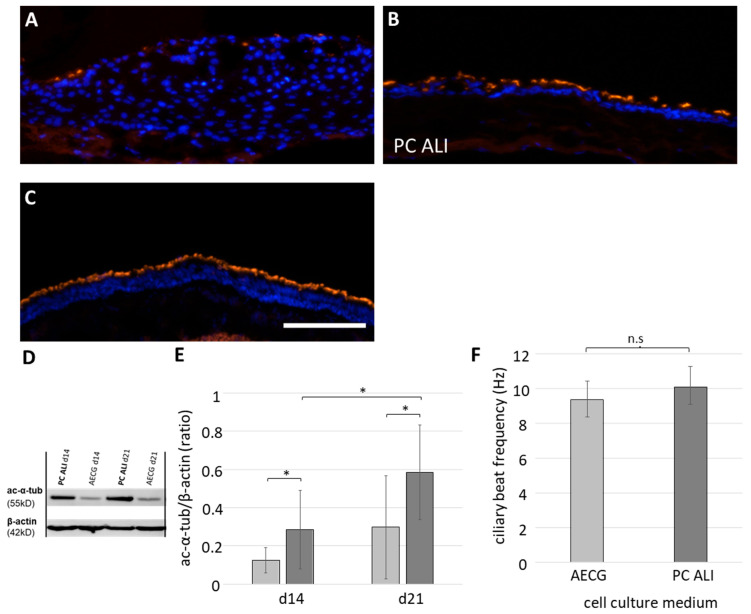
Quantification of the occurrence of cilia and ciliary beat frequency (CBF) in 3D airway tissue models. (**A**–**C**) Cross sections immunostained against acetylated α-tubulin (ac-α-tub, orange) from ALI models cultivated for 21 days with AECG (n = 9 from four donors) (**A**) and PC ALI (n = 6 from four donors) (**B**) and staining of native bronchus (control) (**C**). (**D**) Western blot analysis of ac-α-tub from ALI models cultivated for 14 and 21 days. (**E**) Semi-quantitative protein analysis (AECG (light grey) and PC ALI (dark grey) (n = 5 each, from four donors, with one technical replicate of each sample). (**F**) Measurements of CBF at day 21 (AECG (light grey, n = 8 from three donors); PC ALI (dark grey, n = 7 from three donors)). Nuclei were stained with DAPI (blue). Scale bar: 200 µm. Shown are the mean average with standard deviation. * *p* < 0.05, n.s.: not significant.

To analyze the distribution and quantity of cilia in the models, immunofluorescence stainings against acetylated α-tubulin and Western blot analyses were performed at days 14 and 21 (Figure 5). The functionality of the cilia was determined by ciliary beat frequency (CBF) measurements at day 21. With AECG medium only few apical cilia (Figure 5A) were found, while with PC ALI medium a nearly confluent layer of apical cilia (more than 50% coverage) was present (Figure 5B). Native bronchus also showed confluent layer of cilia (Figure 5C). With PC ALI medium, the amount of acetylated α-tubulin at day 14 and 21 was almost twice than with AECG medium (Figure 5D,E). Only PC ALI models showed a significant increase of the amount of acetylated α-tubulin from 14 to 21 days (*p* = 0.0185). Measurements of the CBF resulted in 9.36 ± 1.06 Hz with AECG and 10.10 ± 1.16 Hz with PC ALI medium (Figure 5F). The latter is similar to the physiological CBF, which was found to be around 10 to 14 Hz [27]. Our data suggested a higher ciliogenesis when cultivated with PC ALI medium and no difference in CBF. 

### 2.5. Evaluation of the Barrier Function

**Figure 6:**
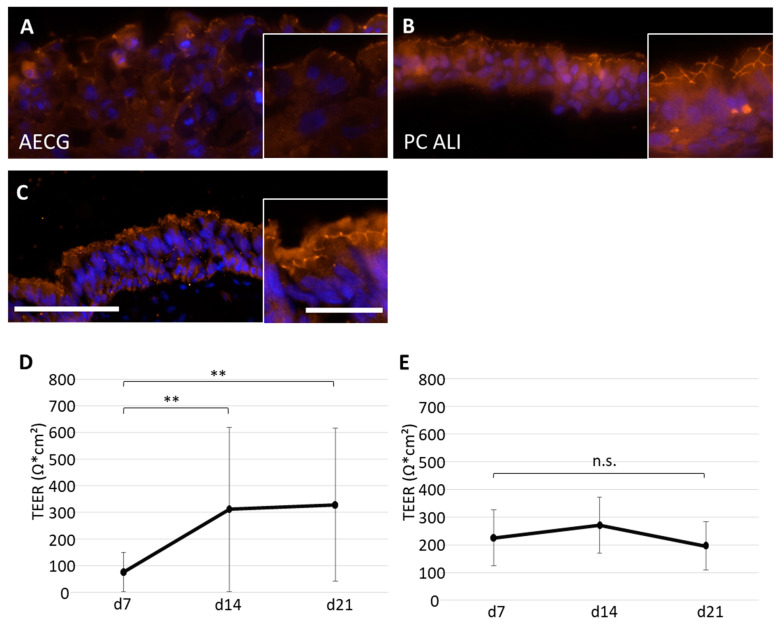
Formation of tight junctions and transepithelial electrical resistance (TEER) measurements in 3D airway tissue models. (**A**–**C**) Immunofluorescence stainings against zonula occludens (ZO-1, orange) on cross sections of 21 days old ALI models cultivated with AECG (n = 9 from four donors) (**A**) and PC ALI medium (n = 6 from four donors) (**B**), and of native bronchus (control) (**C**). TEER values of airway models cultivated for 7, 14, and 21 days, respectively, as ALI culture in AECG (**D**) and PC ALI medium (**E**) from four donors (d7: PC ALI n = 23, AECG n = 33; d14: PC ALI n = 17, AECG n = 33; d21: PC ALI n = 9, AECG n = 20). Scale bar: 200 µm, inserts: 100 µm. Nuclei were stained with DAPI (blue). Shown are the mean average with standard deviation. ** *p* < 0.001, n.s.: not significant.

To examine the barrier function of our airway models, immunofluorescence staining against the tight junction protein zonula occludens (ZO-1) at days 14 (not shown) and 21 (Figure 6A–C) and measurements of the transepithelial electrical resistance (TEER) at days 7, 14, and 21 (Figure 6D,E) were performed. The immunostaining showed a diffuse localization of ZO-1 at day 14 with AECG medium (not shown) and an apical, intercellular localization in less than 50% of the epithelium at day 21 (Figure 6A). In contrast, models cultivated with PC ALI possessed more than 50% of the epithelium apical and intercellular ZO-1 localization at both time points (Figure 6B). Native bronchus showed apical and intercellular tight junctions throughout (Figure 6C). The TEER values increased significantly from 76.12 ± 73.08 Ω*cm^2^ at day 7 to 310.28 ± 307.97 Ω*cm^2^ at day 14, and 328.25 ± 286.09 Ω*cm^2^ at day 21 with AECG medium (d7-d14: *p* < 0.001, d7-d21: *p* < 0.001) (Figure 6D). With PC ALI medium, the TEER values remained quite stable with 224.76 ± 99.88 Ω*cm^2^ at day 7, 271.09 ± 101.31 Ω*cm^2^ at day 14, and 196.03 ± 88.00 Ω*cm^2^ at day 21 (Figure 6E).

## 3. Discussion

In the light of the well-known limitations of both rodent animal models as well as two-dimensional cell culture models, donor-derived primary 3D airway models are becoming increasingly interesting for basic and applied research [10]. The generation of human 3D co-culture models containing airway epithelial cells and donor-matched fibroblasts is still a difficult task. This is explained by the challenging expansion and required mucociliary differentiation of the cells. Here, we report the generation of a 3D airway model with a physiological histological morphology and functioning mucous membrane.

During primary cell expansion, we found that epithelial cells cultivated in PC Ex+ medium grew faster, and a higher cell yield was achieved compared to the culture with AECG medium. We attributed these differences to the varying media composition, however, the exact composition of the PC Ex+ and also PC ALI medium is not disclosed. Therefore, we can only speculate about the growth factor(s) that could make the difference. One possible candidate could be retinoic acid, which is contained in AECG medium at a low concentration of 0.1 ng/mL. It is known that retinoic acid supports airway epithelial differentiation in a dose-dependent manner [28]. A low concentration was associated with the formation of squamous epithelium, while a higher concentration supported pseudostratified epithelium [28]. In line with this, it has been shown that inhibition of the retinoic acid signaling promoted proliferation of distal lung organoids, whereas differentiation was deteriorated [29]. Therefore, one could speculate that PC Ex+ medium does not contain retinoic acid in contrast to the AECG medium. However, the higher proliferation with PC Ex+ medium may also be attributed due to the presence of other growth factors.

Interestingly, almost all hbEC were positive for CK14 when cultured with AECG medium. In contrast, in PC Ex+ medium only one third of cells were CK14 positive. The latter is similar to the conducting airways in vivo, where 40% of the basal cells are CK14 positive [30]. CK14 is found in the basal stem cell compartment in vivo, which is quite heterogeneous, with p63 as a pan-basal stem cell marker, CK5-positive, and CK14-positive cells. P63/CK14-double positive epithelial cells have been reported to be parabasal progenitor cells that are located above the basal cell compartment in vivo [31]. Additionally, CK14 was associated with squamous metaplasia in the distal lung [30]. In AECG medium cultivated 3D models, we also observed a squamous-like morphology with a low number of ciliated epithelial cells and less mucus production, suggesting a defective mucociliary differentiation possibly due to a too low concentration of retinoic acid. In contrast, in PC ALI models, more than 50% of the surface was covered with ciliated epithelial cells, which resembles the in vivo situation with 60–80% of ciliated epithelium [32]. Here, one could speculate that the PC ALI medium contains a significantly higher concentration of retinoic acid than the AECG medium and, therefore, supports the proper differentiation. Further, the epithelial layer thickness was comparable with native bronchial airway epithelium as well [33,34]. By using PC ALI medium, the variability of the epithelial layer thickness, mucus secretion, coverage of ciliated epithelial cells, and TEER values between different donors was considerably lower compared to the 3D models cultivated with AECG medium. In contrast to other published 3D models [21,35], we implemented donor-matched primary fibroblasts and a natural scaffold. In doing so, we took a step forward in the generation of a physiological-like 3D model that is supported by the comparison with bronchial biopsies.

The TEER measurement is considered as standard method to describe epithelial barrier function: The higher the TEER value, the better the epithelial barrier. In our study, 3D models cultivated with AECG medium showed higher and more variable TEER values than models with PC ALI medium. The immunostaining against the tight junction protein ZO-1, however, showed a higher differentiation of tight junctions with PC ALI medium. We, therefore, assume that the higher values with AECG medium are due to the formation of a hyperplastic epithelium with a bigger total surface area, which influences the TEER value, instead of the formation of a tighter epithelial barrier. Similar results have been reported by Yamaya et al., who also found higher TEER values in 3D airway models with squamous epithelial cells [36]. There are several factors known to influence the TEER value [37], which is among others, the composition of the medium. In our study, the differentiation with AECG medium resulted in highly variable TEER values. One reason could be an irregular, not consistent differentiation using AECG medium, which resulted in a varying epithelial layer thickness and tight junction formation. Both have strong influence on the measured TEER values, as discussed before. In vivo TEER measurements of the human bronchial epithelium are not described in the literature to the best of our knowledge. However, the TEER values of PC ALI cultivated 3D models were quite similar to measurements of bronchial rabbit epithelium in vivo in the 1990s, showing values around 266 ± 97 Ω*cm^2^ [38]. Although our more complex 3D models are better comparable with native bronchial tissue than other models, it should be considered that still some limitations exist that includes the limited availability of primary cells, the time and effort, the expenses as well as the donor-to-donor variability.

In the future, our new complex 3D airway model could, for example, serve to model certain aspects of airway diseases like asthma or COPD. At the latter, especially interactions between epithelial cells and their stromal environment, including fibroblasts, play an important role. 

## 4. Materials and Methods

### 4.1. Donor Tissue, Cell Isolation, and Cultivation

Healthy bronchial specimen were obtained from patients undergoing elective pulmonary resection (Table S1). The patient’s informed consent was obtained before surgery and the studies were approved by the ethics committee of the medical faculty of the Otto-von-Guericke-University Magdeburg (vote 163/17, 16 October 2017). hbEC were isolated by cutting the tissue in 1 mm^2^ pieces and plating them with the mucosal side on a petri dish. The tissue was supplied either with AECG medium (PromoCell, Heidelberg, Germany) or PC Ex+ medium (STEMCELL Technologies, Saint Égrève, France) during epithelial cell outgrowth and expansion. For isolation of hbFb, the tissue was cut in 1 mm² pieces and digested overnight in 5 mL DMEM (Gibco, Waltham, MA, USA), 0.75 mL collagenase B (Thermo Fisher Scientific, Waltham, MA, USA) and 50 µL antibiotic, antimycotic solution (Sigma-Aldrich, Darmstadt, Germany). The suspension was filtered first through a 100 µm and then a 40 µm filter and seeded in cell culture flasks, with fibroblast growth medium 2 containing 2% fetal calf serum (PromoCell). hbECs and hbFbs were passaged when a confluency of 80% was reached. The media were changed every two to three days. All cells were maintained under standard culture conditions in a humidified incubator containing 5% CO_2_ at 37 °C.

### 4.2. Generation and Cultivation of 3D Airway Models

For the generation of 3D models, the biological scaffold SIS (decellularized porcine small intestinal submucosa, Fraunhofer Institute for Silicate Research ISC, Würzburg, Germany) [39,40] was fixed between two plastic cylinders (cell crones). First, 1 × 10^5^ hbFb were seeded on the model with fibroblast growth medium 2. The next day, 1 × 10^5^ hbEC were added. We used the hbEC in passage 1 and the hbFb up to passage 4. Cells were expanded in co-culture with AECG or PC Ex+ medium, mixed 1:1 with fibroblast medium. After four days, ALI culture was introduced by removing the apical medium and replacing PC Ex+ with PC ALI medium (STEMCELL Technologies). 3D tissue models were cultivated for 21 days, with three media changes per week.

### 4.3. Assessment of Epithelial Barrier Function

TEER measurements were performed at day 7, 14, and 21 of ALI culture using the cellZscope E (nanoAnalytics GmbH, Münster, Germany). 3D models were transferred to the measurement module and supplied apically with 1 mL and basolaterally with 1.5 mL medium. TEER was determined after 3 h of equilibration in the CO_2_ incubator at 37 °C. Cell-free SIS scaffolds cultured in fibroblast growth medium 2 served as controls (blank values). Blank values were subtracted from the measured TEER values.

### 4.4. Assessment of Ciliary Beat Frequency

After 21 days of ALI culture, CBF of 3D models was measured. Models were washed apically three times with PBS^-^ and cell crowns were removed. The matrix was transferred to a µ-dish (Ibidi GmbH, Gräfelfing, Germany) and covered with 100 µL medium (see above). Videos of ciliary activity were taken with a water immersion objective (NIR Apo 40×/0.8; Nikon, Tokyo, Japan) and a highspeed camera (Genie HM640; Teledyne Dalsa, Waterloo, ON, Canada). From every model, five videos (10 s length) at a frame rate of 100 images/s (640 × 480 pixels) were recorded within 20 min. Within each video, four regions of interest (ROIs) were defined to quantify CBF. CBF was determined by fast Fourier transformation with a MATLAB graphical user interface (Mathworks, Natick, MA, USA).

### 4.5. Histological and Immunofluorescence Staining

The models were embedded at days 14 and 21 and sliced into 10 µm cryo-sections. Mucus production was characterized by alcian blue staining according to standard protocols. For immunofluorescence staining, tissue slices were fixated with 4% paraformaldehyde in PBS^-^ for 10 min and blocked with 3% normal goat or normal donkey serum, with 0.1% Triton X100 (Sigma-Aldrich) in PBS^-^ for 30 min both at room temperature. The samples were incubated overnight at 4 °C with primary antibodies (diluted in blocking serum): polyclonal mouse anti-acetylated-α-Tubulin (1:100, Thermo Fisher Scientific), polyclonal mouse anti-p63 (1:300, Genetex, Irvine, CA, USA), polyclonal rabbit anti-cytokeratin 14 (1:500, BioLegend, Amsterdam, The Netherlands), monoclonal mouse anti-Ki-67 (1:30, Agilent, Waldbronn, Germany), polyclonal mouse anti-Muc5AC (1:100, Thermo Fisher Scientific), polyclonal rabbit anti-Muc5B (1:100, Sigma-Aldrich), polyclonal rabbit anti-ZO-1 (1:100, Genetex). After washing three times in PBS^-^, the tissue slides were incubated for 1h at room temperature with secondary antibodies: Alexa Fluor^®^ 488-conjugated donkey anti-rabbit, Cy™3-conjugated donkey anti-mouse IgG or Cy™3-conjugated goat anti-rabbit IgG (all 1:500, Jackson ImmunoResearch, Ely, UK). Finally, samples were incubated with 1µg/mL DAPI (nuclear stain, Sigma-Aldrich) in PBS^-^ for 10 min at room temperature. The sections were washed three times in PBS^-^, and embedded in Mowiol 4-88 with DABCO (2.5%) (C. Roth GmbH, Karlsruhe, Germany). Images were taken with the Evos Auto FL 2 fluorescence microscope with the objectives Evos 3.1 AMEP4682 (20×/0.4), Evos 1.6 AMEP4683 (40×/0.65), or Olympus Luc 2.7 AMEP4764 (Plan FL N 40×/0.6) (all Thermo Fisher Scientific).

### 4.6. Western Blot Analysis

For semi-quantitative analysis of cilia presence, Western blot analysis of acetylated α-tubulin was performed. Cells were scraped from the matrix and lysed in 300 µL 2× cell lysis buffer (Item J; Sigma-Aldrich) with 3 µL 1:10 diluted Phenylmethansulfonylfluorid (PMSF, Sigma-Aldrich) for 45 min with vortexing every 10 min. The samples were centrifuged at 21.000× *g* and 4 °C for 15 min. The supernatant was frozen at −80 °C. Protein concentration was determined using Pierce BCA Protein Assay Kit (Thermo Fisher Scientific). Protein solutions were separated in a 10% SDS-PAGE (80 V for proteins reaching the separating gel, then 120 V for 120 min). Proteins were transferred to a PVDF membrane in transfer buffer (192 mM glycine, 25 mM TRIS Base, 20% methanol) at 100 V for 90 min on ice. The membranes were blocked in 5% milk powder in tris-buffered saline (TBST) and washed three times with TBST. Anti-acetylated-α-tubulin (1:500) and monoclonal anti-β-actin (1:1000, Proteintech, Manchester, UK) as primary antibodies were diluted in TBST and incubated with the membrane at 4 °C overnight. The membrane was washed three times with TBST. Anti-mouse IgG antibody (Jackson ImmunoResearch) and anti-rabbit IgG antibody (Cell Signaling Technology, Leiden, The Netherlands; both HRP-linked and 1:10,000) as secondary antibodies were diluted in 1% milk powder in TBST and incubated for 1 h at room temperature. The membranes were washed twice with TBST and once with TBS, and then incubated with ECL reagents. The chemiluminescent signals were detected by the ECL Imager ChemoStar (Intas Science Imaging, Göttingen, Germany). Bands were quantified using LabImage 1D (Intas Science Imaging).

### 4.7. Mucin Score and Intraepithelial Mucus Cysts

Apical mucin secretion was analyzed semi-quantitatively. For each 3D model, three different randomly chosen cross sections were evaluated by estimating the amount of immunofluorescence signals of Muc5AC and Muc5B, respectively, per total surface of a cross section. Scoring was: zero points (no detection), one point (sporadic detection on the surface), two points (<50% of the surface), three points (>50% of the surface). (For Muc5B: six models each medium; for Muc5AC: six models with AECG medium, five models for PC ALI medium, all from four donors). The presence of intraepithelial mucus cysts was analyzed using the same cross sections, which were used for the mucin score. Immunofluorescence signals of Muc5AC and Muc5B within the epithelial layer in DAPI-free areas were counted as presence of intraepithelial mucus cysts. Given is the percentage of cross sections on which intraepithelial immunofluorescence signals of either Muc5AC or Muc5B were detected. 

### 4.8. Statistical Analysis

The statistical analysis was performed using InStat (GraphPad Software, San Diego, CA, USA). The Mann-Whitney U test unpaired, two-tailed was utilized for all statistical analyses and represented * = *p* < 0.05 and ** = *p* < 0.01. Except for the TEER measurements, where the Kruskal–Wallis nonparametric Anova Test with Dunn’s multiple comparison test was utilized and represented * = *p* < 0.05 and ** = *p* < 0.01. All data are expressed as mean with standard deviation. For all experiments, the total number of repetitions using how many donors is given in the figure legends.

## 5. Conclusions

The expansion of the primary hbEC in PC Ex+ medium, and the following co-cultivation of the hbEC with donor-matched hbFb on a biological collagen scaffold in a mixture of PC ALI medium and fibroblast medium, allowed mucociliary differentiation and the generation of a physiological-like 3D airway model composed of the epithelial and the stromal component. 

## Data Availability

The data presented in this study are available in this article and the corresponding Supplementary Material.

## Supplementary Materials

The following supporting information can be downloaded at: https://www.mdpi.com/article/10.3390/ijms24044113/s1.
