# Supplementary material for: Optimization of Primary Human Bronchial Epithelial 3D Cell Culture with Donor-Matched Fibroblasts and Comparison of Two Different Culture Media
Source: Int J Mol Sci. 2023 Feb 18;24(4):4113. doi: 10.3390/ijms24044113 (PMC9965758; doi:10.3390/ijms24044113)
Supplement: Supplementary file 1 [file ijms-24-04113-s001.zip › ijms-2134824-supplementary.pdf]

Supplemental Materials

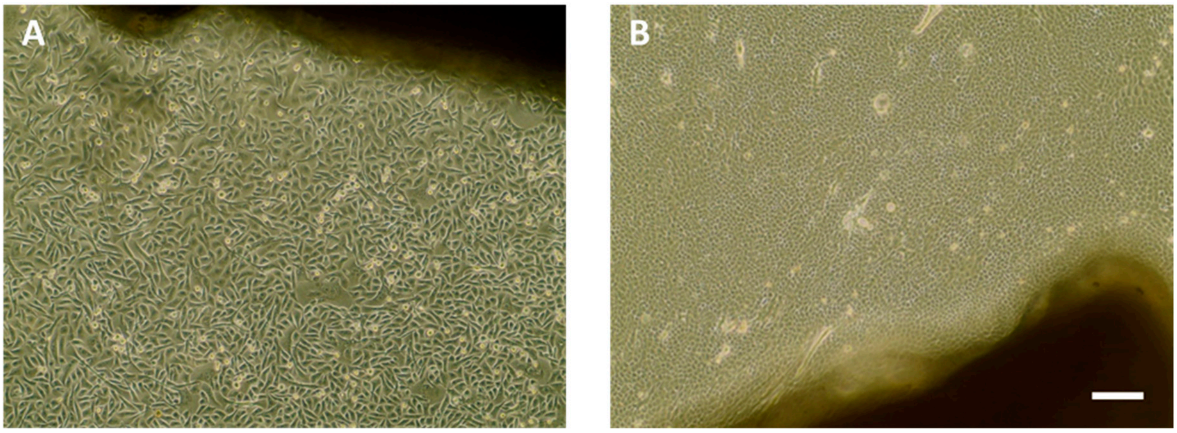

Figure S1: Light micrographs of bronchial epithelial cells (hbEC) growing out of bronchial tissue (black). hbEC were expanded either in (**A**) airway epithelial cell growth (AECG) or (**B**) PneumaCult ExPlus (PC Ex+) medium (each n=6). Scale bar: 200 µm.

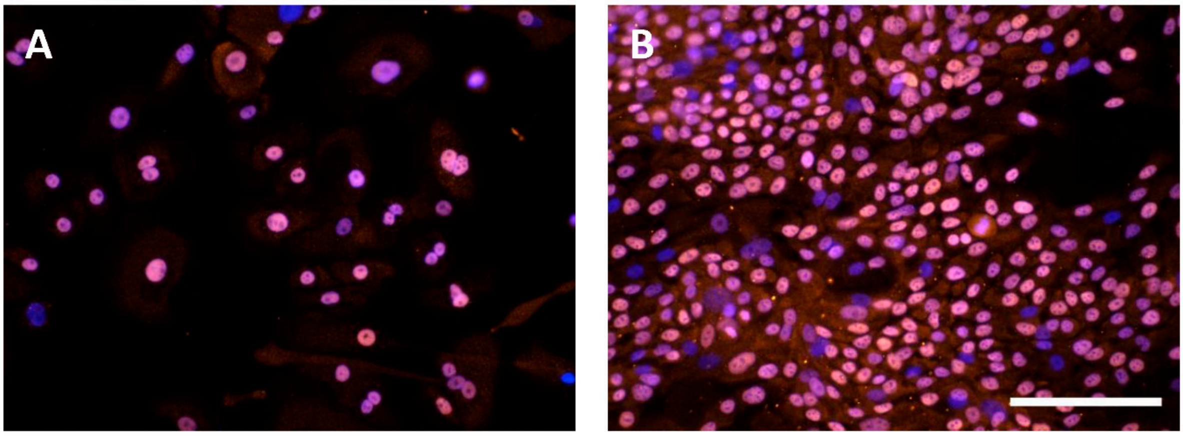

Figure S2. Expansion of primary human bronchial epithelial cells (hbEC). hbEC were expanded either in airway epithelial cell growth (AECG) or PneumaCult ExPlus (PC Ex+) medium and characterized with immunofluorescence staining in passage 1. Immunofluorescence staining against p63 (red) (1:300, Genetex, Irvine, CA, USA) on hbEC cultivated with (**A**) AECG and (**B**) PC Ex+ medium (n = 6 each). Nuclei were stained with DAPI (blue). Scale bar: 100 µm.

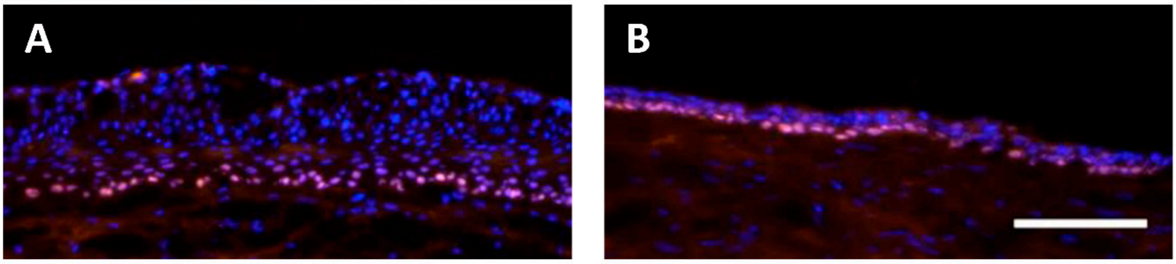

Figure S3. Basal cell layer in 3D airway tissue models. Immunofluorescence stainings against p63 on cross sections of 3D models at 21 days of cultivation with (**A**) AECG (n = 9) and (**B**) PC ALI medium (n = 6). Nuclei were stained with DAPI (blue). Scale bar: 200 µm.

Table S1: Patient characteristics. Bronchial tissue samples were obtained from patients undergoing anatomical lung resection for pulmonary tumors. Informed consent was obtained beforehand.

Nr.	gender	age (y)	BMI	FEV1 (%)	smoking history	pack years	histology	TNM classification	tumor localization	induction therapy	type of surgery
1	male	65	30	88	former smoker	40	pulmonary squamous cell carcinoma	pT1c pN0 L0 V1 Pn0 M0 R0 G2	right upper lobe	no	lobectomy
2	female	81	26	117	never smoker	0	pulmonary adenocarcinoma	pT2a pN0 L0 V0 Pn0 M0 R0 G2	left upper lobe	no	lobectomy
3	male	59	34	60	never smoker	0	pulmonary metastasis of spermatic cord pleomorphic liposarcoma	R0 G3 M1	right middle lobe	no	lobectomy
4	female	31	24	84	never smoker	0	bronchogenic cyst	--	left upper lobe	no	lobectomy
5	male	76	28	112	former smoker	50	pulmonary adenocarcinoma	pT1c pN0 L0 V0 Pn0 M0 R0 G3	right middle lobe	no	lobectomy
6	male	73	34	72	former smoker	55	pulmonary large cell carcinoma	pT1b pN0 L0 V0 Pn0 M0 R0 G3	right upper lobe	no	upper bilobectomy

<u>Abbreviations:</u> y, years; BMI, body mass index; FEV1, forced expiratory volume.
